# Spectral Interferometry with Frequency Combs

**DOI:** 10.3390/mi13040614

**Published:** 2022-04-14

**Authors:** Krishna Twayana, Israel Rebolledo-Salgado, Ekaterina Deriushkina, Jochen Schröder, Magnus Karlsson, Victor Torres-Company

**Affiliations:** 1 Department of Microtechnology and Nanoscience, Chalmers University of Technology, SE-41296 Gothenburg, Sweden; twayana@chalmers.se (K.T.); israels@chalmers.se (I.R.-S.); ekader@chalmers.se (E.D.); jochen.schroeder@chalmers.se (J.S.); magnus.karlsson@chalmers.se (M.K.); 2 Measurement Science and Technology, RISE Research Institutes of Sweden, SE-50115 Borås, Sweden

**Keywords:** laser frequency combs, silicon photonics, optical coherence tomography, spectral interferometry, swept-wavelength interferometry, optical spectroscopy, optical signal processing, optical frequency domain reflectometry, microresonators

## Abstract

In this review paper, we provide an overview of the state of the art in linear interferometric techniques using laser frequency comb sources. Diverse techniques including Fourier transform spectroscopy, linear spectral interferometry and swept-wavelength interferometry are covered in detail. The unique features brought by laser frequency comb sources are shown, and specific applications highlighted in molecular spectroscopy, optical coherence tomography and the characterization of photonic integrated devices and components. Finally, the possibilities enabled by advances in chip scale swept sources and frequency combs are discussed.

## 1. Introduction

Laser frequency combs are coherent light sources formed by evenly spaced optical frequencies whose location in the electromagnetic spectrum can be set with the accuracy provided by atomic frequency references. They have become enabling tools for precision frequency synthesis and metrology [1,2]. Indeed, one of the most remarkable applications of frequency combs lies in the field of optical spectroscopy [3]. In linear absorption spectroscopy, the uniform grid formed by the comb lines directly probes the specimen under test with a frequency resolution fundamentally limited by the comb linewidth. Different instruments have been developed over the last decades that are able to resolve the individual comb lines, including high angular dispersive elements [4], Fourier transform spectrometers with delays matched to the comb’s repetition rate [5] or by multiheterodyne downconversion with another frequency comb that is slightly mismatched in repetition rate [6]. Frequency combs can also be used as rulers against which to calibrate tunable laser diodes [7], including noncontinuously tunable ones [8], and multiple laser diodes can be characterized to cover an ultrabroad bandwidth [9]. Recent efforts in optical frequency-comb based spectroscopy aim at enabling higher acquisition rates in nonlinear spectroscopy [10], measuring emission (fluorescence) spectroscopy signals [11], and advance imaging modalities [12].

Close to absorption spectroscopy is linear spectral interferometry where both the amplitude and phase of an optical sample is retrieved from an interferogram signal under the assumption that the optical source is known. In this tutorial paper, we will give an overview of the field of spectral interferometry using laser frequency comb sources. We begin with a short review of optical frequency comb sources and then give a brief introduction to the basics of spectral interferometry, followed by a discussion on signal characteristics when the broadband source is replaced by a laser frequency comb. We will cover illustrative arrangements, including low coherence, spectral, and comb calibrated swept frequency interferometry. Some examples will be shown in the context of the characterization of passive linear components, highlighting the unique features enabled by the comb source. We will end with a forward looking overview of the field, touching upon advances in chip scale technology as well as a discussion on what future possibilities lie down the road.

## 2. Generation of Optical Frequency Combs

Optical comb sources have evolved tremendously over the last decades (see [2] for an extensive historical overview). The sources that led to the Nobel prize in physics were primarily solid state lasers with repetition rates of few tens of MHz operating in the near infrared. Since then, sources with an amazing diversity of operating wavelengths, performance parameters and footprints have been developed, to satisfy the needs of a large variety of applications. Fiber based frequency combs are commercially available from several vendors and are arguably the workhorse of frequency metrology. They offer significantly improved robustness and ease of operation over their solid state counterparts and have been demonstrated with rates up to a few hundred MHz and phase coherent spectra spanning more than an octave [13] and very low electrical power consumption [14]. However, both solid state and fiber based sources are generally limited to sub-GHz repetition rates, due to the relatively large required cavity length. While there has been some effort to overcome these limitations for solid state lasers [15,16], sources with rates of multiple GHz are typically based on other technologies. One such system are electro-optic comb generators that are based on the electro-optic modulation of a continuous wave laser using a microwave signal provided by an RF oscillator (see [17,18] for an overview). The flexibility of this approach, both in terms of center wavelength and frequency-spacing, has made these electro-optic combs popular versatile sources for many applications. However, the high driving voltages required to generate broad bandwidths and the noise limited by RF oscillators means that they have largely been relegated to applications with only modest bandwidth and stability requirements, although reference [19] showed that these limitations can be overcome to generate an octave spanning f−2f stabilised electro-optic comb. Much of the optical frequency comb development has been driven by experiments in research laboratories and most sources are dependent on benchtop size, power hungry and expensive equipment. Extending applications beyond research laboratories toward consumer applications requires the development of cheap, miniaturised devices with low power requirements. In the last decade, we have, therefore, seen a concentrated effort to develop photonic chip comb sources, by taking advantage of advanced photolithography and nanofabrication technology. The two arguably most prominent examples sources are: Kerr microcombs, which rely on parametric mixing inside a high-Q cavity nanofabricated in a nonlinear optical material [20], and integrated semiconductor mode locked lasers [21]. Other approaches are resonant [22] and nonresonant [23] electro-optic comb generation in thin film Lithium Niobate. We refer the interested reader to the recently published comprehensive review of integrated comb sources in [24].

## 3. Basics of Spectral Interferometry

The technique known as Fourier transform spectroscopy (FTS) dates to the 1950s [25], decades later than the development of the Michelson interferometer. FTS benefited from advances in computation. Fourier transform spectroscopy stands out today as a workhorse for astronomical observation, and as a diagnostic tool in the biological and medical sciences [26]. The interested reader is referred to [27,28] for more comprehensive and detailed overviews of the topic.

**Figure 1:**
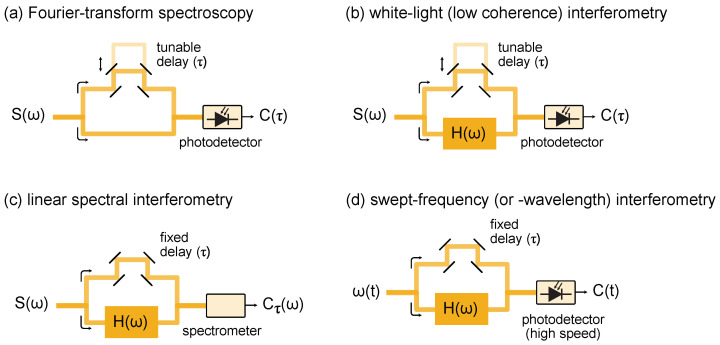
Simplified schemes for Fourier transform spectroscopy and spectral interferometry with broadband light sources. (**a**) Fourier transform spectroscopy; (**b**) low coherence (or white light) interferometry; (**c**) linear spectral interferometry and (**d**) swept frequency interferometry. In Fourier transform spectroscopy (**a**), an interferogram is recorded as a function of the relative delay between the arms of an interferometer (a Mach–Zehnder in the figure). By Fourier processing the interferogram, it is possible to retrieve the optical power spectrum of the light source, S(ω). If the spectrum of the light source is known, this technique can be used to characterize, in amplitude and phase, a component under test, which is mathematically represented by a complex transfer function H(ω). The interferogram can be recorded in different ways. In white light interferometry (**b**), the signal is recorded as function of the delay between arms. In linear spectral interferometry (**c**), the delay between arms is fixed, and the interference pattern is recorded in the spectral domain using an optical spectrometer. An alternative modality for spectral interferometry is based on a coherent source with a single optical frequency that is (ideally) linearly swept over time. An interference pattern over time is recorded with the aid of a photodetector and analog to digital converter unit.

A Fourier transform spectrometer records the interference pattern of an optical source as it travels through different arms in an unbalanced interferometer (see Figure 1a). An interferogram signal, C(τ), arises from the time integrated power as the relative delay between the arms, τ, is scanned

(1) C(τ)∝∫S(ω)dω+Re∫S(ω)exp[−iωτ]dω.

**Figure 2:**
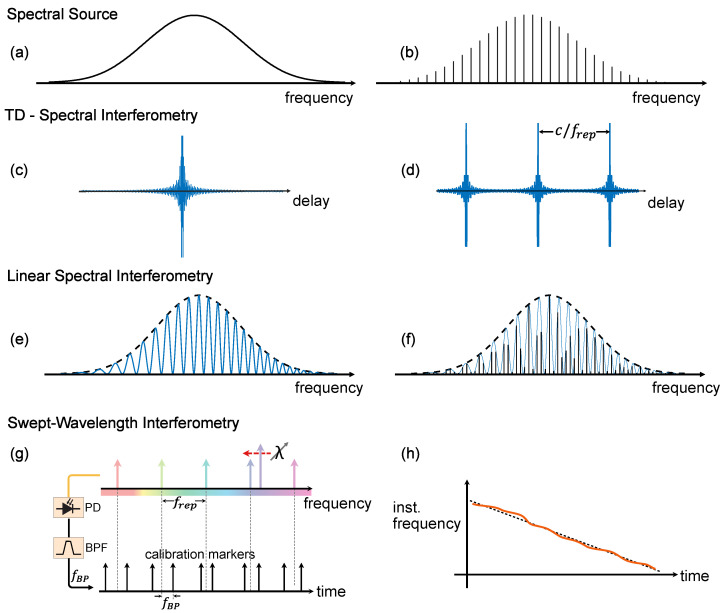
Comparison between spectral sources and the interferometric signals obtained with different configurations. (**a**,**b**) Spectral sources. (**c**,**d**) Time domain-spectral interferometry (TD- SI). (**e**,**f**) Linear spectral interferometry. (**g**,**h**) Frequency comb assisted laser calibration for SWI. Broadband sources are exemplified with a continuous (**a**) and discrete nature (**b**). The interferogram using a TD approach based on white light is single burst when the difference of the optical path is zero (**c**). In contrast, if a frequency comb is employed, the interferogram pattern consists of several bursts spaced by $c/f_{rep}$ (**d**). The interference signal using an SDI approach is measured with a CCD camera when using a conventional source (**e**) and discrete distribution of spectral components is recorded when using a frequency comb (**f**). In (**g**), a sweeping laser is heterodyned against the frequency comb of repetition rate $f_{rep}$. Calibration markers are detected every time the scanning laser is $\pm f_{BP}$ (central frequency of the bandpass filter) away from the comb line. Beats notes are unevenly spaced for the laser being swept nonlinearly. (**h**) Calibrated instantaneous laser frequency (orange trace) on the top of linear approximation (dotted line).

The (unknown) optical power spectrum of the light source, S(ω), is obtained by Fourier processing the interferogram. FTS is insensitive to the spectral phase of the light source and, hence, cannot distinguish between spectrally incoherent (Figure 2a) and coherent light sources (Figure 2b). The spectral resolution in FTS is given by the longest achievable delay. This can be an issue because of the difficulties in aligning the moving arm over long distances. Note that a Mach–Zehnder interferometer, as depicted in Figure 1a, is not the only possible arrangement and, in fact, FTS is regularly implemented in a Michelson interferometer. FTS requires the independent calibration of the delay, typically implemented by counting the interference fringes of an auxiliary interferometer using a laser with a known emission wavelength. The interferogram produced using a FTS spectrometer is depicted in (Figure 2c,d) when using incoherent and a coherent source, respectively.

Low coherence (or white light) interferometry is a slight modification of FTS as showed in Figure 1b. Here, the light source is known, and used to retrieve the complex transfer function of a device under test, H(ω). The interferogram signal as a function of the delay becomes

(2) C(τ)∝∫S(ω)[1+∣H(ω)∣2]dω+2Re∫S(ω)H(ω)exp[−iωτ]dω

This technique was widely used in the 1980s for the characterization of the dispersion in optical fibers [29]. Advances in supercontinuum generation sources have enabled the precise characterization of higher dispersion terms in photonic crystal fibers [30]. Optical coherence tomography (OCT) is another practical application of low coherence interferometry, where the device under test is an optically transparent sample of biological tissue [31]. A broadband light source acts as a coherence gate, allowing to measure backscattered reflections with an axial (depth) resolution given by the inverse of the source bandwidth. The dynamic range of the measurement is above 100 dB, allowing to retrieve signals with a penetration depth of a few millimeters, which is a challenging feat for other microscopy modalities due to the large scattering in biological tissue.

Linear spectral interferometry is represented in Figure 1c. It differs from low coherence interferometry in that the relative delay between the interferometer arms is fixed and the interferogram is recorded directly in the frequency domain with an optical spectrometer. The interferogram signal (Figure 2e) in this case is

(3) Cτ(ω)∝S(ω)[1+∣H(ω)∣2]+2Re[S(ω)H(ω)exp[−iωτ]]

As the delay between arms is fixed, this configuration is more robust and simpler to set up. The spectral resolution here is provided by the resolving power of the spectrometer itself. By Fourier processing the interferogram, it is possible to retrieve the amplitude and phase of the transfer function of the device under test. Inverse Fourier transformation of Equation (3) yields three terms, at t = 0, −τ and +τ. The time delay must be chosen such that it is long enough to avoid the temporal overlap of these signals. The signal peaking at either +τ or −τ could then be filtered and its inverse Fourier transform provide the complex information of H(ω) [32].

In the context of OCT, spectral interferometry implemented with a grating-based and CCDs provide an enhancement in sensitivity and signal to noise ratio, compared to time domain OCT [33]. This has been exploited in situations with low light levels or that require high refresh rates. One potential drawback is that the limited number of pixels yields a fundamental tradeoff between spectral bandwidth (affecting the axial resolution) and frequency resolution (affecting penetration depth).

Swept-wavelength interferometry, Figure 1d, is a submodality of spectral interferometry, where the broadband light source is replaced by a frequency tunable laser (also known as a swept wavelength laser). The laser frequency is swept back and forth in a quasitriangular fashion, covering a large optical bandwidth. This approach results in better sensitivity than linear spectral interferometry and capitalizes on advances in rapidly tunable lasers [34]. While the bandwidth covered by swept-wavelength lasers lies below what is available from the superluminiscent diodes and supercontinuum light sources commonly used in broadband spectral interferometry, the spectral resolution in this modality is improved because it is only limited by the instantaneous linewidth and sampling rate of the receiver. The comb assisted laser calibration (Figure 2g,h) in the context of swept-wavelength interferometry enables high precise spectroscopy.

## 4. Frequency Combs in Spectral Interferometry

This section will describe a few techniques where frequency combs enable or support spectral interferometry.

### 4.1. Time Domain Interferometry

As discussed previously, the information of either an unknown spectrum or sample can be retrieved using FTS and white light sources in combination with Michelson interferometers. These techniques are indifferent to the degree of coherence of the spectral source. However, using a broadband coherent light source brings benefits such as collimated long distance propagation with high spectral brightness, high signal to noise ratio and high resolution [5,35,36]. The good spatial coherence of an optical frequency comb offers the possibility to be coupled to an enhancement cavity to improve the sensitivity of the spectrometer. The efficient injection of light to the enhancement cavity is achieved by mode matching its free spectral range (FSR) with the repetition rate of the comb [37].

**Figure 3:**
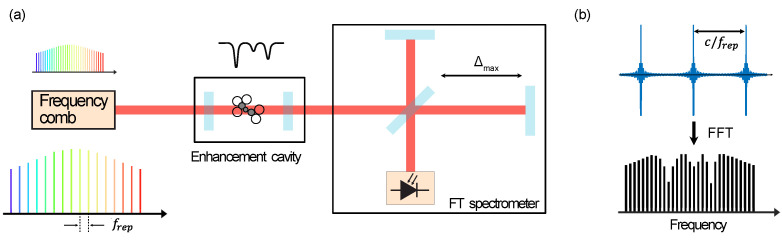
Setup of a comb based FTS. (**a**) A frequency comb with a repetition rate $f_{rep}$ interrogates a sample contained in an absorption cell, an enhancement cavity can be used to increase the effective absorption length. The light enters a Michelson interferometer where a measurement arm is translated over a scanning range $\Delta_{max}$ to produce and interferogram, (**b**), that consists of several bursts spaced by $c/f_{rep}$. The Fourier transform of the interferogram yields the absorption spectra of the sample under test.

Fourier transform spectrometers using frequency combs have been implemented using a movable delay, as shown in Figure 3a. A usual configuration is to acquire a double sided interferogram, i.e., with an equal delay at both sides of the zero path position. In this manner, the signal to noise ratio (SNR) is increased by $\sqrt{2}$, since the data is collected at each side of the central burst; furthermore, the phase correction is avoided in this configuration.

In a first implementation, the white light source was replaced by a mode locked (MLL) laser coupled to a rapid scan Fourier transform spectrometer [38]. In this work, the spatial coherence offered by the frequency comb was exploited to increase the signal to noise ratio (SNR) by two orders of magnitude, when comparing with a thermal source. Although the measurement of broadband spectra with sensitive acquisition was demonstrated, the individual spectral lines were not resolved because the resolution was still limited by the maximum path length achievable by the interferometer.

Since the frequency comb emits a series of short pulses, the interferogram produced with the comb based FTS consists of a periodic modulation pattern repeating at integer multiples of $c/f_{rep}$, where *c* is the velocity of light and $f_{rep}$ is the repetition rate of the comb (see Figure 3b). Therefore, to resolve the individual spectral lines, the interferogram should be measured over a path length that is at least equal to $c/f_{rep}$ [5,35,39]. In [39], this is achieved by scaling the optical delay line to increase the resolution of the spectrometer, allowing to observe several correlation patterns per delay scan. Another scheme to fully resolve the lines of the combs was demonstrated in [5], by matching the interferogram length with the repetition rate of the comb so that the scanning range $\Delta_{max}=c/f_{rep}$. This, in practice, means sampling one point per optical mode.

One practical aspect in FTS is that the recording of the interferogram is always truncated, due to the finite distance of the scanning mirror. Therefore, the interferogram is windowed by the transfer function of the acquisition method. In the Fourier domain, the spectrum of the interferogram is convolved with an instrumental line shape (ILS), which, generally, is a sinc function when the temporal acquisition is a boxcar. This broadening is detrimental when the width of an absorption line of interest is narrower than the nominal resolution of the instrument, since the lineshape measured will resemble the ILS. In [5,40], this limitation was overcome using a specific approach of sampling the data that allowed to set the nominal resolution of the instrument. It is shown that, when the nominal resolution is exactly the same as the repetition frequency, the ILS contributions at the neighbouring optical modes becomes zero. This allows to sample the comb modes without being affected by the ILS disturbance [5]. Furthermore, typical undesired effects, such as ringings, are avoided, since the frequencies of the comb are aligned with the FTS sampling points.

Since the main limitation in a mechanical FTS is the scanning range $\Delta_{max}$, using a comb based setup allows to perform high resolution measurements only limited by the comb mode width [41]. These previous advancements have demonstrated that a high resolution spectra is achievable without the need of extending the optical path difference in FTS. However, the dependence of the nominal resolution of the instrument with the repetition rate is still present and is reflected in the need to have optical path differences in the order of meters for combs spaced by hundreds of MHz.

Sensitivity can be further increased by cavity enhancement (CE), where the frequency comb is coupled with a high finesse cavity to enhance the interaction of the light with the sample [42]. Cavity enhanced frequency comb spectroscopy using FT spectrometers has been reported to increase signal to noise ratio with short time acquisition measurements [43,44]. An important challenge in CE-FTS is the need of locking the comb modes to the cavity modes to prevent the cavity acting as a frequency filter. This can be implemented by using a two point PDH locking technique [45]; in this approach, the frequency to amplitude (FM-AM) noise conversion is the main source of noise in CE-FTS. In [46], the noise is reduced by using a home built FTS with an autobalanced detection, achieving a shot noise limited performance, spectra recorded with an SNR above 30 dB and a resolution of 380 MHz within a measurement time around 400 s. Later work employed noise immune cavity enhanced optical frequency comb spectroscopy, where the entire comb is locked to the enhancement cavity and modulated to produce sidebands at a modulation frequency that is commensurate to a multiple of the cavity FSR [36,47].

### 4.2. Spectral Domain Interferometry

In linear spectral domain interferometry (SDI), the spectrometer is based on an angular dispersive element (e.g., grating) and a CCD camera. Spectral domain interferometry results in higher sensitivity, SNR and speed than time domain white light interferometry but it has a limited spectral resolution. Spectral domain interferometers use a wide spectral range source to produce spectral interference patterns. Using a frequency comb, the total interference spectrum is created by the interference signal of the individual spectral lines of the comb recorded with a spectrometer. In this case, the spacing of the interference pattern is spaced by $c/f_{rep}$ due the pulsed nature of the comb.

**Figure 4:**
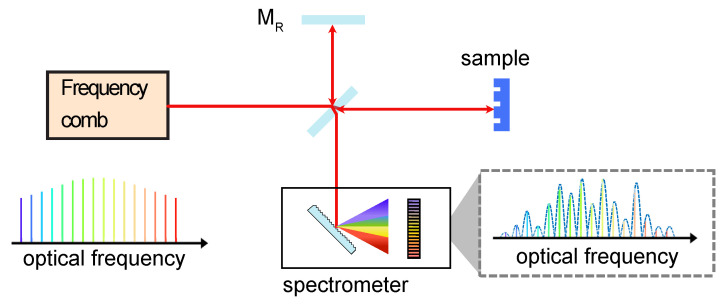
White light interferometer using a frequency comb. The light of the comb is split in two parts using a beam splitter, then is directed to the fixed reference mirror (M${}_{R}$) and the surface of the sample. The reference and measurement beams are recombined to produce the interference signal detected by the CCD’s spectrometer.

The setup of a spectral domain interferometer is shown in Figure 4. In contrast to the time domain approach, the delay is fixed in this configuration. As seen in Figure 2e, the phase of the spectral interference varies with respect of the frequency, which, in combination with Fourier analysis, allows to retrieve parameters such as the geometrical path difference of the interferometer or the thickness and group refractive index of materials [48,49,50]. Another benefit of using a comb in SDI is the long coherence length over a broad bandwidth that allows long distance measurements [51]. The measurable distance range can be extended, since the nonambiguity range is limited by the coherence length of the modes and not to the inherent 2π phase ambiguity of conventional interferometers [49,52].

A major drawback in employing spectral domain interferometry is the finite resolution of the CCD’s spectrometer. In the context of optical coherence tomography, a variety of techniques, such as phase-shifting [53] and interpixel shifting to double the number of samples [54], have been reported to enhance the spectral resolution and sampling rate. However, these methods rely on mechanical sweeping of the reference mirror and the translation of the detector, respectively, compromising the acquisition speed of the system. Since the light in an SDI setup is spatially spread over an array of photo elements, the interference fringes are convolved with a rectangular function that represents the width of an individual CCD pixel. Therefore, a denser pattern of the spectral fringes suffers an amplitude suppression caused by the limited resolution of the detector. In Bajraszewski et al. [55], the use of a passive frequency comb was proposed to replace the continuous light source. In this manner, the sensitivity is enhanced since the resolution is defined by the linewidth of the individual comb line instead of the pixel size and interpixel crosstalk is also avoided. In this particular case, the spacing between the comb lines does not match consecutive pixels, therefore, the number of samples is reduced, which is detrimental for the intended OCT application. The spectrum sampling is then performed by slightly tuning the repetition rate of the comblike source, thereby increasing the number of samples recorded and improving the imaging range. Since the shifting of the spatial position of the frequency lines rely on the FP cavity length tuning, the consecutive combs are not shifted equidistantly in the CCD array which produces imaging artifacts. In later work, the FP interferometer was replaced with an all fiber Sagnac loop interferometer to overcome the channel spacing, still offering a high depth sensitivity and high axial resolution [56].

In OCT systems, the high axial resolution is as important as maintaining high axial scan (A-scan) speeds, in order to provide the video rate imaging needed in medical applications. This, at the same time, poses a challenge in terms of data acquisition. For real time imaging, multi-MHz A scan rates are needed in order to image large volumetric fields, therefore increasing the computation needs. Circular ranging OCT (CR-OCT) has emerged as an alternative of the linear ranging, by combining a frequency comb with a ranging system that discriminates highly sparse signals [57,58]. In contrast to linear ranging, this approach alleviates the acquisition bandwidth requirements, since the interferometric signals are folded and the sparsity is handled to reduce the number of measurements. Linear ranging is performed using a continuous source of light where the interference pattern is modulated depending on the physical delay, which results in a higher modulation frequency with a longer delay [59]. This is not the case with circular ranging, where the fixed space between the frequency components of the comb produces a single low frequency modulated signal for all physical, equally spaced, reflected signals. In this way, the measured delay contains the superimposed information of multiple depth locations. Later implementations of this approach focused on improving the correction of the dispersion [60], aliasing artifacts [61] and recovering the absolute delay positions of the sample [62].

The work in [58] not only exploits the frequency comb properties but it also combines it with the retrieval of complex interference fringes using quadrature detection [53,63] and a stretched pulse technique. Fourier domain mode locking (FDML) has been extensively studied as a frequency swept laser, therefore enabling higher imaging depths with A scan speed rates comparable to SS-OCT systems [64,65]. The application of such frequency comb swept lasers will be discussed in detail in Section 4.4. Frequency combs not only offer equally spaced frequency components to improve the resolution of spectral OCT, but also offer increased signal sensitivity due their spectrally distributed power. With the advancement of frequency comb generation, microcombs were studied as a light source for spectral OCT, exploiting their broad bandwidth when operating in the chaotic state [66,67]. Supercontinuum generation on a chip scale scale has been also explored for OCT application, achieving a sensitivity up to 105 dB with a low pump power [68]. Coherent frequency combs generated on chip in the current spectral OCT systems could potentially offer a broader bandwidth and better noise performance [67,68]. Furthermore, on chip microcombs could be utilized in a circular ranging scheme to perform optical domain-subsampling with a less complex architecture than that proposed in previous works.

### 4.3. Comb Calibrated SWI and OFDR

**Figure 5:**
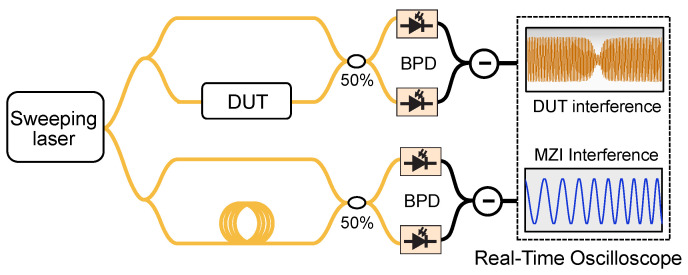
Schematic experimental setup of SWI of a DUT. Auxiliary interferometer (without DUT) assists in tracking and compensation of laser’s sweep nonlinearity.

Swept-wavelength interferometry (SWI) has become a widespread high precision measurement technique that is applied in the characterization and measurement of photonic devices [69,70] and optical fibers [71,72,73,74,75], as well as in OCT [76,77]. In the simplest case, it relies on an interferometric structure based on a broadband sweeping laser where one of the arms contains a device under test (DUT), see Figure 5. The laser source is swept across the measurement range in the interferometer and the signal is then detected by photodetectors and digitized by an analogue to digital converter. Subsequently, it is possible to extract parameters of interest, most commonly, the transfer function of the DUT or the time domain impulse response, which is simply the FFT of the transfer function. As the frequency (wavelength) is tuned during the measurement scan, the received signal is frequency dependent. The frequency of the laser, in turn, changes with time, and, hence, is time dependent.

One of the main challenges in SWI is that the laser sweep is never perfectly linear in time. Deviations from a purely linear wavelength sweep cause significant measurement errors and broaden the impulse response function [72]. This limitation is usually overcome by introducing an auxiliary interferometer to trace the nonlinear tuning of the laser [72,78,79]. It is then possible to convert the nonlinear wavelength sweep to a linear frequency dependence by extracting new sample points from the received signal of this additional interferometer, as long as the laser sweeps in continuously. An alternative approach uses the signal from the interferometer as a clock to trigger a real time oscilloscope, which avoids the potentially large number of interpolations required for resampling [80,81,82]. If the relative delay between the two arms in the auxiliary interferometer is $\tau_{t}$, then the period of the output pattern of the additional interferometer will be $\;\Delta \nu_{t}=1/\tau_{t}$. Using this signal as an external clock to sample the fringe pattern output by the measurement interferometer enables sampling at equal optical frequency steps $\;\Delta \nu_{t}$. Instead, the active linearization of the ultrabroad-band tunable laser sources using the self-heterodyne interferometer has been illustrated for high resolution laser radar application [83]. However, the dispersion and other systematic effects of the interferometer cause deviation in the linearity.

The conventional laser calibration lacks absolute accuracy and suffers from a systematic error caused by a wavelength dependent fiber group delay. It requires calibration of the of the delay arm and operates in a stable condition [80,84]. To aid this calibration, one can use a comb as a frequency ‘ruler’. Precise and accurate selfreferenced frequency combs have been applied for the wavelength calibration of a tunable laser source in broadband microresonator spectroscopy [7,9]. Its relevance for the nondestructive characterization of the spectral phase response of the ultra-low loss photonic devices was demonstrated in [85]. In addition, a tunable laser calibration with a free running mode-locked laser in absolute distance measurement [86,87] and imaging [88] has been demonstrated. As only the repetition rate is stabilized, this approach needs the sweeping laser to tune faster than the drift in carrier envelope offset frequencies (CEO).

**Figure 6:**
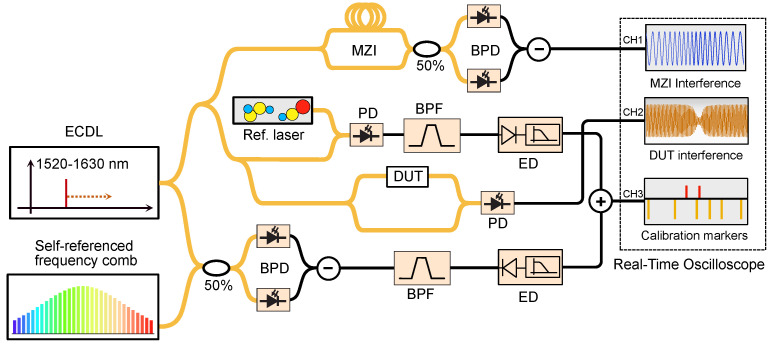
Frequency comb calibrated swept wavelength interferometry. Measurement setup for the acquisition of frequency calibrated SWI/OFDR interference fringe pattern. Frequency calibration is achieved through beat markers provided by fiber selfreferenced frequency comb, a laser locked to a known absorption resonance and the fiber MZI interference pattern. The acquisition of patterns in the boxes of an oscilloscope are from different channels. ECDL: external cavity diode laser; BPD: balanced photodetector; PD: photodetector; BPF: bandpass filter; ED: envelope detector; PD: photodetector [85].

The schematic diagram for frequency comb assisted swept wavelength interferometry is shown in Figure 6a. It uses a selfreferenced frequency comb as an optical ruler in combination with a Mach–Zehnder interferometer to calibrate the nonlinearity of the laser on the fly. In principle, a single optical frequency comb would be enough for calibrating the optical frequency axis, as the laser is swept in frequency. Frequency comb modes are defined by the pulse repetition rate ($f_{rep}$) and carrier envelope offset frequency ($f_{ceo}$) as $f_{c}=nf_{rep}+f_{ceo},n\in N$. The equidistant frequency lines are used to generate the beat notes with the tunable laser. A narrow bandpass filter of central frequency $f_{BP}$ generates a calibration marker when the scanning laser is $\pm f_{BP}$ apart from the closest comb line. Therefore, the unknown laser frequency is calculated as $f_{l}=nf_{rep}+f_{ceo}\pm f_{BP}$. The sign of the $\pm f_{BP}$ term is related to the direction of the laser scanning. The peaks of the reference and calibration markers are traced by envelope detectors biased with opposite polarity. A reference laser locked to a molecular transmission line whose optical frequency, $f_{ref}$, is stable and known with an accuracy better than half the repetition rate (with the aid of, e.g., a wavelength meter) facilitates the calibration of the frequency axis by resolving the mode number $n=f_{ref}-f_{ceo}/f_{rep}$. Note, however, that some applications do not require absolute accuracy but the correction of the nonlinear sweep of the laser. This calibration procedure assumes the laser tunes at a constant rate between consecutive sets of calibration markers separated by the repetition rate. However, for repetition rates in the order of 100 MHz and larger, this is not the case. One could decrease the repetition rate of the comb by, e.g., synchronized external modulation. This solution introduces other difficulties, such as a reduced optical power per line and encompassed SNR in determining the calibration marker. The nonlinear sweep rate of the laser in between calibration markers is traced more precisely with the aid of MZI with an FSR below the repetition rate of the comb [85,89].

The phase difference (Δϕ) measured between the calibration markers with the MZI and the corresponding frequency difference (Δf) referring to the comb lines enables calculating a local group delay τ=Δϕ/(2πΔf). It is assumed to be constant for calculating the relative laser frequency $f_{rel}\left(t\right)=\Delta \phi \left(t\right)/\left(2\pi \tau \right)$ between markers. Then, the absolute frequency is estimated referring $f_{rel}\left(t\right)$ to one of the markers. The precision of the laser calibration is limited by the bandwidth of the BPF and the memory depth of the acquisition unit per trace.

The interferometric characterization of the DUT can take two distinct arrangements: swept wavelength interferometry (SWI) and optical frequency domain reflectometry (OFDR). The disparity lies in how the interference signal carries the optical properties of the DUT. In SWI, a fraction of the laser power probes the DUT in a transmission configuration and interferes with the reference signal at a coupler. Conversely, distributed backscattered light from the DUT interferes with the reference signal in OFDR. The residual dispersion of the interferometer arms can be calibrated in the absence of DUT. With regards to the auxiliary interferometers, these could be co-integrated on the same chip as the DUT [70]. This results in enhanced stability, but does not resolve the systematic error introduced by the dispersion. Reference [85] measures the response of the DUT connected in one arm of the imbalanced interferometer in a single scan. In particular, integrated microresonators and waveguides as DUT have distinct impulse responses corresponding to the interferometer virtually without DUT and with the DUT. It enables retrieving the corresponding complex transfer functions by taking the Fourier transform and, thus, the DUT spectral response (detailed in Section 5.4).

### 4.4. Fourier Domain Modelocked Lasers

**Figure 7:**
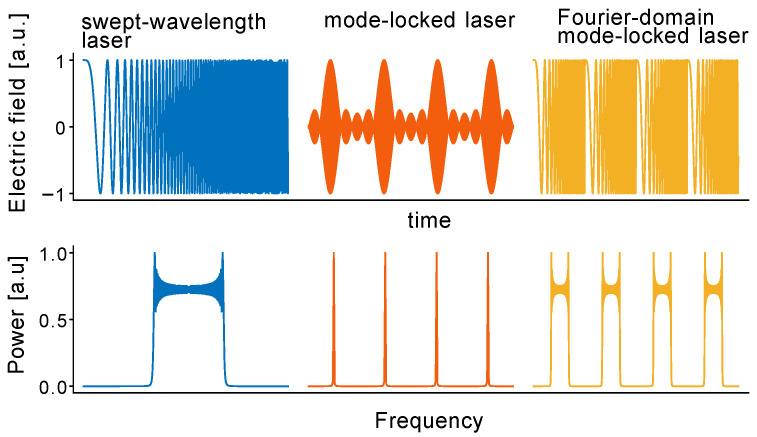
Electric fields (**top**) and intensity of the spectra (**bottom**) of a swept wavelength laser, a standard mode locked laser (MLL), and an Fourier domain mode lock (FDML) laser.

The standard configuration of a tunable laser is based on an external cavity containing a tunable bandpass filter [90]. The wavelength of the optical filter is tuned in order to sweep the laser’s wavelength and sufficient time is needed to allow lasing in the transmission bandwidth to build up from spontaneous emission. This leads to limitations of the sweeping speed and output power, as well as broadening of the linewidth and concomitant shorter coherence time [77]. These problems can be mitigated by extending the laser cavity and periodically driving the optical bandpass filter, considering that its sweep rate is synchronized with the optical roundtrip time of the lightwave in the cavity. In this regime, called Fourier domain mode-locking (FDML), light from one frequency sweep travels through the cavity and returns to the optical bandpass filter precisely at the moment when the transmission of the filter is at the initial spectral position [64]. The entire optical sweep is stored inside the cavity and, therefore, it is not necessary to generate lasing from amplified spontaneous emission every time [64]. In principle, this enables a relatively fast sweep rate (e.g., a bandwidth of 143 nm at 1050 nm wavelength was scanned at a rate of 1.67 MHz in [91]) that is useful for applications in spectroscopy and OCT [92] (see Section 5). Ideally, consecutive frequency sweeps have the same phase and are mutually coherent. There is almost no energy loss in the optical bandpass filter because the backcoupled light contains only frequencies that are matched to the transmission window of the filter at each moment. In the frequency domain, this indicates that all longitudinal modes that are not transmitted through the narrowband filter at a particular time experience destructive interference. Thus, the phases of the longitudinal modes must be locked. Standard mode locked lasers (MLL) have longitudinal modes locked with constant phase, which corresponds to the generation of a train of short pulses at a repetition rate equal to the cavity round trip time. FDML lasers, on the other hand, have modes locked with a different phase relationship. The laser output, therefore, is not just a sequence of short pulses, but a train of frequency sweeps which constitute highly chirped long pulses, as shown in Figure 7. The tunable narrowband filtering, in this scenario, is equivalent to an infinite number of narrowband amplitude modulators that are slightly out of phase.

However, there are several problems when working with standard FDML lasers. One of them is that the coherence length is limited by the bandwidth of the fiber Fabry–Perot filter (FFP-TF) and significant drops in sensitivity can be observed when the ranging depth increases [93,94,95,96]. To improve the sensitivity over a longer ranging depth, several approaches suggesting the use of frequency combs have been proposed [55,56,97]. Another problem is that the frequency sweep is not ideally linear and it is necessary to resample the interference fringe pattern in order to have the data evenly sampled in the frequency domain before applying the FFT. This usually requires an additional interferometer in the measurement system [92,93], which is computationally expensive and limits real time operation in experiments. One of the successful solutions for linearizing the FDML sweep was to incorporate the second and third harmonics of the drive waveform to FFP-TF [95]. This method, however, demands that the FFP-TF is thermally stable and has a high frequency response.

To further improve laser based measurement systems, Tsai et al. proposed to incorporate a fixed narrow band frequency comb fiber Fabry–Perot (FFP-FC) filter inside the cavity of conventional swept lasers and FDMLs [65], called a frequency comb (FC) swept laser. The FFP-FC in the laser cavity slices the swept signals in the frequency domain into discrete comb lines, with identical spacing given by the FSR of the comb filter. It effectively improves the coherence length and narrows the instantaneous linewidth of the swept laser [65,98]. However, it is limited by the finesse of the FFP-CF. In [99], instead of a FFP-FC filter, the discretization of the FDML laser is implemented with an on chip high Q microresonator. The adoption of the microresonator filter narrows the instantaneous linewidth of the FC laser down to 1.5 GHz and is accompanied by an improvement in coherence length. As the FFP-TF is swept in time, the laser generates a set of fixed frequency steps with varying amplitudes. Depending on the values of the bandwidth of the FFP-TF $\Delta \nu_{FFP-TF}$ and the FSR of the FFP-FC $FSR_{FFP-FC}$, two scenarios exist. If $\Delta \nu_{FFP-TF}<FSR_{FFP-FC}$, the laser will generate only a single frequency at a time and the intensity output will be strongly modulated. On the other hand, if $\Delta \nu_{FFP-TF}\approx FSR_{FFP-FC}$, the laser can operate in a superposition of FFP-FC frequencies and is partially modulated in intensity. It is necessary to operate the first regime, where the bandwidth of the FFP-TF is less than the FSR of frequency comb FFP-FC, in order to generate frequency steps and to produce a stable modulation in the output intensity trace. Frequency comb swept lasers outperform conventional swept lasers in sensitivity. As was demonstrated in [65], a frequency comb swept laser can achieve a −5 dB sensitivity roll off using a recalibration with Mach–Zehnder interferometer and −1.2 dB sensitivity roll off using selfclocking, which is comparable to or better than tunable lasers. However, as a downside, the demonstrated sweeping speed of 100 kHz in [65] cannot compete with the tuning speed of FDML lasers and some state of the art ECLs [100]. Another nuance that should be also considered is that an interferometric signal that has a frequency higher than one half of the sampling rate will be aliased. The FSR of the FFP-FC $FSR_{FFP-FC}$ defines the number of samples that can be generated as the laser is swept across its tuning range. Hence, it is important to choose the $FSR_{FFP-FC}$ so that the frequency comb spacing is small enough to provide a large enough measurement depth range and sufficient number of samples during the frequency sweep. As a future outlook, it can be considered to calibrate the FDML laser with the frequency comb, similar to what has been performed in [85].

### 4.5. Dual Comb Interferometry

**Figure 8:**
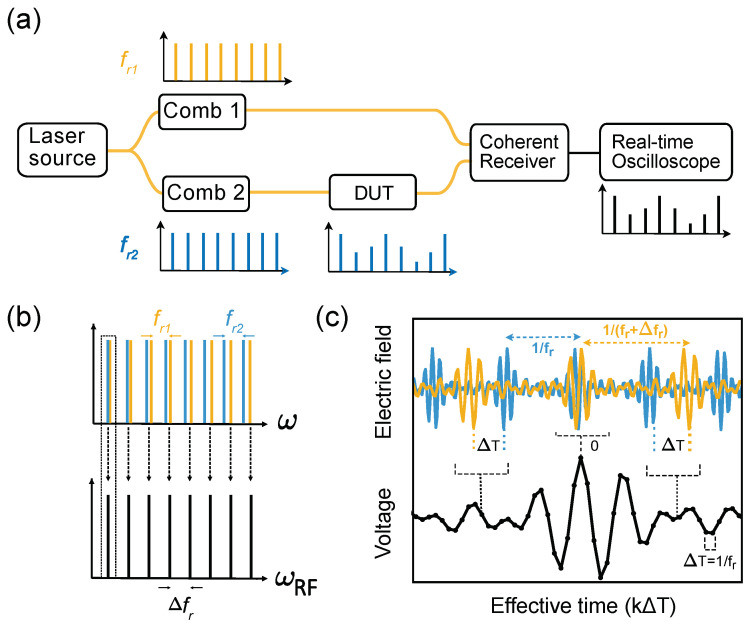
Frequency domain description of DCI: (**a**,**b**) Two frequency combs with slightly different repetition rates, $f_{r1}$ and $f_{r2}$, interact with each other and at least one comb passes through the DUT, whose frequency response changes the shape of the comb lines. The resulting absorption and phase on the comb teeth are modified into the corresponding amplitude and phase of the measured RF comb (green) with the repetition frequency $f_{r2}-f_{r1}=\Delta f_{r}$. The RF spectrum can also be obtained by Fourier transforming a series of time domain interferograms. Time domain description of DCI: (**c**) Two pulse trains with slightly different repetition rates, $f_{r}$ and $f_{r}+\Delta f_{r}$, beat each other in a photodetector. These two optical pulse trains interact and effectively ’walk through’ each other. The resulting photoreceiver signal below is a product of two comb pulses integrated over the receiver bandwidth and the signal can be viewed as an interferogram. This output is usually a function of the effective time, kΔT, where *k* is the sample number at time intervals of ΔT. The large central part of the received interferogram corresponds to the simultaneous arrival of the two pulses (when ΔT is 0). A weak ringing visible on the ’tails’ contains absorption information of the DUT.

In the previous sections, we discussed how spectroscopic information can be retrieved by scanning a mechanical delay arm or the laser frequency. Alternatively, an additional comb can be used to perform the scanning. This is known as dual comb spectroscopy/interferometry (DCS/DCI) and has been reviewed in detail in the literature [6,18]. Briefly, DCS maps information from the optical to the RF domains, when two combs of slightly different repetition rate interfere (see Figure 8).

One of the favorable points of such an arrangement is that it can be used for the characterisation of different DUTs. In this case, one of the combs has to pass through a DUT before beating in a photo-detector in order for the absorption lines to be visible in the RF domain. By analyzing the resulting interferograms, different properties of the DUT can be extracted. This is also the principle behind equivalent-time sampling techniques, where, usually, a train of narrow pulses are used to ‘walk through’ the signal under test.

When detecting broadband periodic signals, the *equivalent-time sampling* technique is powerful. The idea was pioneered by Janssen [101,102] in the development of electrical oscilloscopes in the 1950s and already applied to the optical domain in 1968 by Duguay and Hansen [103]. The key is to use short sampling pulses to cut portions from the periodic signal. If the repetition rate of the sampling pulses is slightly detuned from that of the signal, the subsequent samples will sample different portions of the signal, which then can be detected at a much lower rate. In fact, this is mathematically equivalent to the process of dual comb spectroscopy in the time domain, since the Fourier transform of the signal and sampling pulses are two frequency combs with different repetition rates. The principle is the same as the one used in stroboscopes, which can ‘freeze’ or decelerate a rapid periodic event to something that can be observed with the human eye.

A crucial part in the sampling is the optical gate mechanism which needs to be broadband and nonlinear, since the output is the product of the sampling pulses and the signal. Before the advent of dual comb interferometry, various optical nonlinearities have been used for optical sampling, e.g., a Kerr gate [103], fiber four wave mixing [104], a nonlinear crystal [105] or a fiber loop mirror [106]. Since silica based optical fiber has an extremely clean and broadband a nonlinear response it has been a popular choice for optical sampling systems. Especially after the development of highly nonlinear fibers in the early 2000s [107], where fiber four wave mixing was again used. An alternative to these is the so called ’linear sampling’ scheme [108], where the sampling pulses are used as a local oscillator in a homodyne detection scheme. This scheme also has the benefit of being able to detect both the phase and amplitude of the signal, in contrast to the nonlinear schemes that only detect the intensity of the samples. An obvious drawback, however, is that the sampling pulses and the signal must be sufficiently close in frequency to enable homodyne detection. An overview of these techniques can be found in [109]. Later work, similar to these works but focused on spectral phases rather than temporal intensities, have used DCI to retrieve the spectral phase of a second comb [110].

The advent of self-referenced frequency combs facilitated the stabilization of the two degrees of freedom of an MLL (line spacing and frequency offset). This allowed revisiting the concept of linear optical sampling by establishing mutual phase coherence between two frequency combs with slightly detuned repetition rates [111] and the development of DCI. Maintaining mutual phase coherence over a long acquisition time enables the coherent averaging of subsequent interferograms and improving the signal to noise ratio of the interferometric signal. Advances in digital signal processing today allow for performing DCI with free running lasers [112].

DCS can be realized with different comb sources. While the early years utilized femtosecond mode locked laser frequency combs, in recent years, electro-optic frequency combs [17] have been widely used in dual comb spectroscopy. EO combs do not rely on a cavity for attaining mode spectrum, and hence provide flexibility in setting the center frequency and line spacing in an independent manner. Since EO combs are fed by a continuous wave laser, they can easily attain mutual phase coherence in a DCI arrangement [110,113,114,115]. DCI with EO combs have been used for a wide range of applications, including spectroscopy [116], vibrometry [117], and imaging [118].

Another type of implementation involves sweeping the comb repetition rate by tuning the RF source [119,120]. The comb is split in two arms and a delay can be placed in one of them so that when the repetition rate of the EO comb is swept, the output is similar to that obtained with two slightly different repetition rate combs. Such a setup is equivalent to a dual comb scheme with two mutually coherent frequency combs, in the sense of the speed and resolution, and offers significant experimental simplification and cost savings. One of the experimental issues is that the delay line introduces a phase modification only in one MZI arm, which can impact the mutual coherence. This, however, can be solved by putting a phase compensator after the delay line [120]. Another potential drawback is that the acquisition speed is limited by the tuning speed of the RF oscillator, rather than the detector’s bandwidth, as in the case of conventional DCI. Depending on the choice of the RF clock, this can decelerate the measurement speed.

In the most standard configuration, the SNR per spectral element in a thermal noise limited configuration is [121]

(4) $$SNR\approx \frac{\sqrt{\tau_{DAQ}n_{comb}}}{n},$$

where $\tau_{DAQ}$ is acquisition time and $n_{comb}$ is the number of detected comb photons per second. However, the real SNR per spectral element will depend on the dominant noise source [121]. It becomes apparent from the above equation, that there is a complex trade-off between SNR, acquisition rate and number of lines. Combs with large line spacing, such as EO combs [122], microcombs [123] or integrated MLLs [124], can be configured with repetition rates in the order of GHz, resulting in SNR up to 50 dB per line. DCS schemes implemented with large repetition rate combs enable very fast acquisition rates, at the expense of spectral sampling resolution. Depending on the purpose of experiments, it is possible to use different comb configurations with various characteristics including number of lines and repetition rates.

Measurements can also be carried out when the static DCI is complemented with the wavelength tuning of the CW laser source, in order to simultaneously measure the intermediate frequencies over the full comb bandwidth [125,126]. Such a strategy overcomes the fundamental trade-off between acquisition speed and sampling frequency resolution. Another advantage is that it is not necessary to use an auxiliary interferometer for the laser’s sweep nonlinearity compensation, since it is possible to eliminate these distortions by the subtraction of the averaged nonlinear phase component extracted from all comb line scans. The method, however, employs complicated digital signal processing (DSP) steps for coherent stitching to produce a broadband transfer function [126,127].

### 4.6. Optical Arbitrary Waveform Characterization

**Figure 9:**
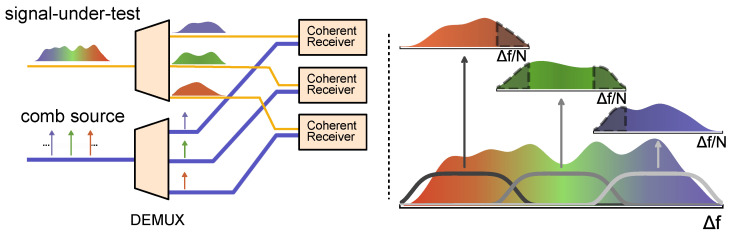
Principle of optical arbitrary waveform characterisation through spectral slicing. (**Left**): A broadband signal under test is demultiplexed using a wavelength division demultiplexer and detected using the lines from a frequency comb source. (**Right**): Phase and amplitude alignment principle. The sub-bands have an overlap region (shaded spectrum) due to the finite response of the demultiplexing filters.

A drawback of the equivalent time sampling systems described in Section 4.5 is that they cannot achieve single shot measurements of an arbitrary optical waveform. While this limitation can be overcome by constructing a set parallel optical samplers with exact time delays [128], such a system can be difficult to manage. We will, instead, focus the discussion in this section on an alternative approach that is often denoted as optical arbitrary waveform characterisation through spectral slicing. This technique has the capability for the single shot characterisation of a completely arbitrary optical waveform with a bandwidth much larger than the detecting electronics. Spectral sliced optical waveform characterisation became feasible with the advent of coherent communication systems and the resulting development of high bandwidth coherent detectors, and real time sampling oscilloscopes with rates of 10 s to hundreds of GS/s and was first demonstrated by Fontaine et al. [127]. The principle behind the technique is depicted in Figure 9. The broadband signal under test is spectrally sliced into a number of narrow sub-bands using a wavelength multiplexing device such as an arrayed waveguide grating (AWG). A frequency comb operating at a repetition rate corresponding to the frequency spacing of the AWG is then separated into its individual lines, each centered close to the center frequency of one of the sub-bands of the sliced signal under test. Each sub-band is then detected inside a coherent detector, where the comb line with the same center frequency acts as the local oscillator. The full broadband input waveform is then reconstructed by realigning the relative phases and amplitudes of the individual slices to each other in a DSP step. The processing relies on the finite impulse response of the AWG windows, which results in a small spectral overlap between the slices, as well as the phase locked nature of the comb. Thus, by traversing through the spectral slices and aligning two neighboring slices to a common phase and amplitude value in their overlap regime, it is possible to fully construct the input waveform with extremely high fidelity.

The linewidth and frequency stability of the detector comb can detrimentally affect the measurement. However, the primary factor that limits measurement performance is the received SNR. The SNR is largely determined by the electronics in the setup. The modern analog to digital converters (ADCs) with sampling rates of 80 GS/s used in these measurements have an effective bit resolution quantified by the effective number of bits between 4 and 5.5. This corresponds to an SNR of around 25–35 dB, which can impose limitations, particularly at the sub-band edges used for phase and amplitude alignment. The system is, therefore, typically not limited by comb performance, unless the power per line (or, equivalently, the optical SNR after amplification) is not so low that the equivalent SNR is lower than the one given by the electronics.

The original demonstration relied on a 40 GHz electro-optic frequency comb and discrete components to characterise a signal with up to 160 GHz bandwidth, which was later extended to cover 228 GHz [129]. Most recently, detection of a 320 GHz signal was demonstrated using a SiN microcomb [130].

## 5. Practical Applications

This section will exemplify and discuss some of the applications of the techniques described above. We will go through molecular spectroscopy samples, characterization of optical fibers, integrated photonic devices, optical coherence tomography, and optical signals for use in optical communications.

### 5.1. Molecular Spectroscopy

Frequency comb capabilities, such as absolute frequency calibration over a broad spectral range, have been employed in different configurations for understanding the complex phenomena of molecular dynamics [131,132]. As referred in Section 4.1, FTS has been widely used for this purpose, where the frequency comb not only offers a wide spectral coverage but high resolution leveraged by the individual modes linewidth. Depending on the configuration, frequency combs can offer precise frequency calibration measurements with high sensitivity, high SNR and fast acquisition speeds.

Molecular spectroscopy in the mid-infrared is especially attractive, since most of the strong vibrational transitions are present in this spectral region. Recent demonstrations of DCS in the mid-infrared have been reported for the characterization of fundamental CH stretch in molecules [133], recovering transmission and dispersion spectra of atmospheric gases in the 2–5 μm region and absorption spectra of CO${}_{2}$ and NH${}_{3}$ using optical parametric oscillators [134,135] as comb generators. However, DCS in the mid-infrared is still challenging because the generation of frequency combs in this spectral range mainly relies on nonlinear frequency conversion methods [135,136,137,138,139]. Therefore, most dual comb spectroscopy implementations have concentrated in the near-infrared region, since direct emission from mode locked lasers is readily attainable. MLLs have been the workhorse of DCS for the study of ro-vibrational overtones of intermolecular phenomena. One of the main challenges in DCS is attaining a mutual coherence in both combs; the work in [140] introduced an arrangement of master–slave combs with a feed forward control to correct the carrier envelope offset frequencies [140]. Broadband absorption spectroscopy was presented in [141], where accurate molecular line measurements of methane and acetylene were performed, only limited by Doppler broadening. Double resonance spectroscopy (DRS) is an established method to obtain Doppler-free spectra, where CW lasers are used in a pump–probe configuration to excite molecules and probe the excited transitions [142]. In [143,144], a frequency comb is used as a probe in a DRS setup to measure sub-Doppler transitions of molecular species in the near infrared. The utilization of a frequency comb enables a broad spectral coverage of sub-Doppler transitions currently limited by the bandwidth of tunable lasers used as a probe. A similar configuration is implemented in [145], where an electro-optic comb probes the cesium transitions generated with a fast switched pump laser, with a microsecond scale provided by the time resolution of the comb.

It has been also shown that FT spectrometers in combination with frequency combs can be used to measure molecular spectra with absorption linewidths narrower than the nominal resolution, as discussed in [5,40]. The work by Maslowski et al. [5] uses a mechanical FTS (similar to Figure 3a) with a subnominal resolution to parallelly measure entire molecular bands at near- and mid-infrared. By solving the fundamental limitation of the spectral resolution, this work demonstrates that high resolution spectra can recorded without large instrumental complexity. This is similar to coherent averaging in DCS, where the two combs are locked to an RF reference which allows to synchronize the temporal acquisition.

DCS has also been widely used to study the spectra of atmospheric gases, replacing conventional FTS spectrometers [146,147]. A quantitative study of two dual comb spectrometers with similar characteristics to measure greenhouse gas concentrations in the near infrared in an open path configuration was reported in [148]. The gas flux concentrations were measured over a 2-week period in outdoor conditions, with the retrieved data by the two instruments being one order of magnitude lower than other similar spectroscopic techniques [147,149].

The use of trains of femtosecond pulses for the study of nonlinear interactions of molecules has been extensively studied [150,151,152,153]. In impulsive stimulated Raman scattering (ISRS), a pump–probe configuration is used to address molecular transitions to produce a coherent high frequency shifted signal [154]. ISRS can also be implemented in combination with DCS, whereby a pulse from one comb coherently excites the vibrational level of a molecule, while the second comb with a different repetition rate then probes the sample at different times [10,155,156,157]. Other nonlinear interactions have also been used together with DCS, e.g., four wave mixing based spectroscopy [158].

The area of comb based molecular spectroscopy contains many more examples than the above which are mainly devoted to dual comb based schemes. A more in-depth review can be found in [159,160].

### 5.2. Characterization of Optical Fibers

Characterization of optical fibers is usually devoted to the extraction of attenuation, group delay and dispersion, both in single mode and multimode fibers. In the latter, effects of linear mode coupling and mixing also come into play, both for polarization and waveguide modes. Using measurements of the transfer function, it is possible to represent a spectral amplitude/phase profile of the fiber under test. These results can be used for improvements of fiber optic link design, or in the modeling of properties in more complex optical fibers, such as multicore and coupled core fibers [161,162]. An early example of comb based fiber characterization is [163], where a dual quadrature detection scheme based on polarization demultiplexing was used to characterize the dispersion of the fiber under test.

DCS is especially favorable for measurements of small phase variations, which can be challenging to detect in short fibers. This opportunity was investigated in [110], where a spectral phase measurement of pulses dispersed by propagation over 20 km of optical fiber was presented. The dispersion coefficient of the single mode fiber was estimated as D=16.5 ps/km/nm, consistent with the known dispersion of the standard single mode fiber. In [122], the dispersion of a 19.9 m single mode fiber was measured. Using measured sequences of interferograms, the authors retrieved the quadratic spectral phase introduced by the fiber along the C band and, after parabolic fitting, estimated the group velocity dispersion parameter of the fiber under test, which was calculated to be $\beta_{2}=-21.93\pm 0.02$ ps/km, in good agreement with the dispersion parameter for standard single mode fibers.

Most recently, DCS was employed as a tool in phase sensitive optical time domain reflectometry (ΦOTDR), which is a well established technique that assists in spatiotemporal measurements of environmental parameters in fiber. In this method, highly coherent pulses propagate inside a fiber under test and experience elastic scattering, such that the back reflected signal, after being detected and time resolved, provides a measurement of variations in the temperature or strain along the fiber length. The main drawback of the ΦOTDR is that the process requires detection bandwidths in the GHz range in order to obtain high spatial resolutions, which increases the cost and complexity of the setup. In [164], the authors added an EO comb to the experimental scheme to interrogate the fiber and sample the back scattered light. The EO comb was accompanied with a coding of its spectral phase that avoids the formation of high peak power pulses. The comb signal backscattered by the fiber interfered with the second comb with the same spectral phase coding and was coherently detected in a dual comb configuration. This measurement configuration leads to a substantial gain in the SNR when compared to single pulse ΦOTDR, but manifests a straightforward decoding that encompasses only basic processing operations. Moreover, the photo detected signal required to retrieve the response of the fiber has a duration that is orders of magnitude longer than the period of the probe signal. This time expansion is a consequence of the spectral downconversion that takes place in the DCS. Therefore, it eases the requirements for receivers used in the setup and spatial resolutions in the centimetre range can be achieved with detection bandwidths in the MHz range or lower.

**Figure 10:**
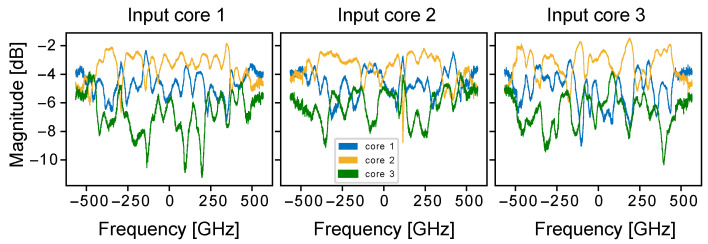
Stitched magnitudes of the transfer function extracted from 45 spectral comb windows with light entering the different cores in the respective graphs, and the exit cores are shown in different colors [126].

As mentioned in Section 4.5, DCS has some limitations related to the discrete nature of the spectral comb lines. Particularly, narrow band variations that fall between the comb lines or delays that are larger than the inverse repetition rate of the combs, cannot be measured using the static DCS. These issues can be overcome by using a tunable laser source that is swept over a comb line spacing, thus combining the DCS and SWI [125]. This technique effectively increases the sweeping rate of the laser by N times, where N is the number of comb lines, while providing improved spectral resolution over DCS. DC-SWI can be very effective with measurements of the coupled core fiber that require a rapid technique, since the coupling coefficients vary rapidly in the ms time scale. Such measurements have been demonstrated with a 1.6 km three coupled core fiber [126], where the transfer function was extracted and differential mode group delay values were estimated. Figure 10 shows the obtained magnitude of the three coupled core transfer function over the 1.1 THz comb bandwidth. Each plot corresponds to a different input fiber core, thus providing a 3×3 transfer function for the fiber under test.

### 5.3. Optical Coherence Tomography

Optical coherence tomography (OCT) is a successful interferometric technique developed to perform noninvasive imaging of biological samples [31]. There are two main approaches to acquire the axial structure of a sample depending on the detection schemed used, namely, time domain (TD)-OCT and Fourier domain OCT (FD-OCT). Similar to Fourier transform spectrometers, in TD-OCT the acquisition is performed by sweeping an optical delay line, which means the signal is recorded as a function of time delay. Conversely, in FD-OCT, the raw interferometric data is recorded in the Fourier domain, and the sample imaging is performed after the Fourier transformation of the wavelength dependant reflections [165].

FD-OCT advancements have surpassed TD-OCT, offering higher sensitivity, acquisition speeds and SNR [33,166,167]. One of the variants of the FD-OCT scheme is implemented using a broadband light source, a spectrometer and a CCD camera to record the data [168]. Another approach is the so called swept source OCT (SS-OCT), where a CW laser is tuned over a broad range using a high speed photodetector [76,166]. SS-OCT can provide a higher acquisition rate due to the utilization of high speed photodiodes instead of line cameras. However, the axial resolution in SS-OCT is still below what Spectral-OCT systems offer, due to the limited bandwidth provided by tunable lasers. Another limitation in SS-OCT is the sweeping stability of the laser. The use of Fourier domain mode locked lasers have attracted significant interest to overcome the stability and sweeping range; however, the imaging range is still limited by the coherence length of the signal due to dispersion effects in the laser cavity [64,169,170]. Indeed, superior coherent length can be achieved by reducing the instantaneous linewidth of the source. In this context, a frequency comb (FC) that offers precise frequency spacing and narrow instantaneous linewidth is highly relevant. In addition, the discrete frequency steps inherent to the FC enable precise real time recalibration of the OCT interference fringe. The improvement in coherent length or imaging depth using the FC in FDML is compared with other different sources in [65,99].

### 5.4. Spectroscopy Analysis of Microphotonic Devices

SWI and OFDR have become standard techniques for the nondestructive analysis of integrated photonic devices [80,85]. Obtaining access to spatial features and spectrally resolved amplitude and phase information facilitates the diagnosis of fabrication errors and improved designs [171]. Using a frequency comb as a ruler against which to calibrate the tunable laser results in absolute accuracy and retrieving the dispersion profile with precision over a broad bandwidth [7,9,85]. In the context of interferometry, the precise laser calibration enables removing an inevitable nonlinear tuning effect from the interference pattern. This is performed by mapping the pattern into an equally spaced optical frequency.

**Figure 11:**
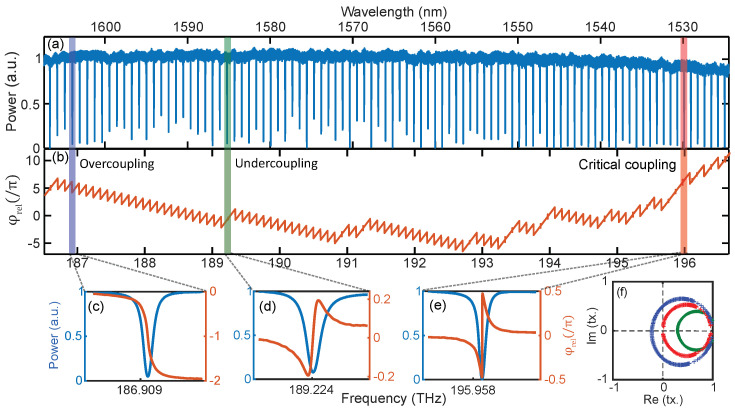
(**a**) Normalized transmission scan of microresonator. (**b**) Linearized effective phase response of the resonator. Different coupling conditions: (**c**) Overcoupling. (**d**) Undercoupling. (**e**) Critical coupling. (**f**) Resonances in the complex plane. The color of the plots corresponds to the resonances marked by the same color bars [85].

Figure 11 presents a recent example where the complex transmission spectrum of a high Q silicon nitride microresonator is analyzed with comb calibrated SWI. Not only the frequency and location of the resonances can be retrieved (and, hence, the microresonator dispersion) but also the phase profile within each and every resonance frequency. This allows for determining unambigously the coupling condition, which can otherwise be affected by coupling between different transverse mode families. In the SWI of the microresonator, the transmission response modulates the envelope, and the phase response is encoded in an oscillation of the interference pattern. The interference pattern also includes the response of the unbalanced arm. The transfer function of this umbalanced arm ($H_{ref}$) is extracted and removed from the overall transfer function ($H_{tot}$). This results in the complex transfer function of the cavity $H_{ring}=H_{tot}/H_{ref}$ (more detail in [85]). The broadband normalized transmission spectrum and the phase response are shown in Figure 11a,b. Magnifications of the retrieved phase and the measured magnitude spectra for different coupling conditions are presented in Figure 11c–e. The phase profiles in Figure 11c,d indicate the resonances are in over couple and under couple regimes, respectively. In Figure 11e, the resonance location has zero transmission and an abrupt π-phase transition, which is indicative of critical coupling. The complex transfer function of these resonances are represented in the complex plane (Figure 11f). The loop traces anticlockwise as the phase proceeds from the positive to negative value. The point crossing the real axis corresponds to the resonance location and its sign is determined by the cosine of the resonance phase value.

**Figure 12:**
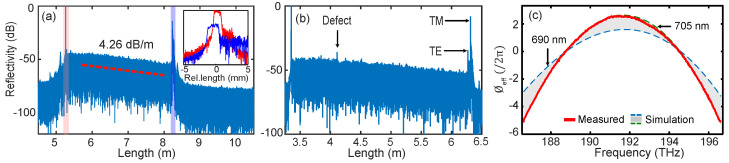
OFDR of the waveguides. (**a**) Spatial reflectivity of fundamental quasi-TE mode (inset: magnification of reflection peaks). (**b**) Spatial reflectivity of fundamental quasi-TM mode. The waveguides are identical in geometry but configured with different fiber delay lengths. (**c**) TE mode measured and simulated effective phase response plot normalized by 2π [85].

Comb calibrated OFDR has also been used recently for measuring the distributed losses of integrated waveguides [85]. In OFDR, the spectral and spatial properties of the DUT are embedded in the distributed reflection of the probe signal. The resampling of the interference in optical frequency and its inverse Fourier transform generates a spatial reflectivity profile or impulse response (Figure 12a,b). The Fourier transform of this spatial distribution results in the complex transfer function ($H_{tot}\left(\omega \right)$). By considering the waveguide as a multilayer reflecting medium, $H_{tot}\left(\omega \right)$ is expressed as:

(5) $$H_{tot}\left(\omega \right)=\prod_{j}H_{j}\left(\omega \right)e^{-i\omega \tau_{j}}$$

where, $H_{j}$ is the transfer function and $\tau_{j}$ is the delay of the DUT arm with respect to the reference arm. Figure 12a,b has two dominant reflection peaks attributed to the Fresnel reflection from the front and rear facets. From Equation (5), the transfer function corresponding to these two peaks are represented as $H_{ref}\left(\omega \right)e^{-i\omega \tau_{ref}}$ for the front and $H_{wg}\left(\omega \right)e^{-i\omega \tau_{wg}}$ for the end facets. Here, $\tau_{ref}$ represents the wavelength dependent optical path length delay virtually with no waveguide and $\tau_{wg}$ with the waveguide. Therefore, the effective spectral phase response of the waveguide is $\phi_{eff}\left(\omega \right)=\omega \left(\tau_{wg}-\tau_{ref}\right)$. The measured phase profile of the waveguide is the red curve shown in Figure 12c. The convex parabolic symmetry of the phase refers to the anomalous GVD of the waveguide. The numerical simulation of the waveguide considering a 10 nm fabrication uncertainty has a good agreement with the measurement in Figure 12c (gray pattern). The phase response attributed to the waveguide dispersion is calculated using the Taylor expansion of the propagation vector and the roundtrip length ΔL of the waveguide as $\phi_{\mathrm{eff}}\left(\omega \right)=\left(\beta \left(\omega \right)-\beta_{0}\left(\omega_{0}\right)-\beta_{1}\left(\omega_{0}\right)\left(\omega -\omega_{0}\right)\right)\times \Delta L$.

The reflectivity profile in between the reflection peaks is associated with the propagation loss of the waveguide. The propagation loss is wavelength dependent per se but it is assumed to be constant over the scanning range in the OFDR. Therefore, the waveguide propagation loss is measured by applying a first order polynomial fit (Figure 12a red line), which gives a loss of 4.26 dB/m. As OFDR is highly sensitive to point reflections, it can precisely spot small defects in the waveguide. In Figure 12a, there is no spurious peak along the waveguide section, which indicates no fabrication defect in the tested sample (Figure 12a). In Figure 12b, however, a tiny reflection peak is observed along the waveguide section. In addition, it has a residual TE mode reflection peak attributed to the power coupling from the TM mode along the propagation. The waveguide fabrication is based on stitching error compensation between adjacent writing fields reported in [171].

### 5.5. Characterization and Detection of Photonic Signals

As described in Section 4.6, frequency combs can be used in a variety of ways in the detection and characterization of broadband optical waveforms, where the bandwidth can be much higher than the available electrical bandwdith. We will mention a few examples in this section.

A key application for waveform sampling has been the monitoring of signals in fiber communications. For example, in 1994, Takara et al. [105] used optical sampling to monitor and detect eye diagrams for a 100 Gb/s time division multiplexed signal, and 500 Gb/s was demonstrated in [172]. The sampling system was also used to study the asymmetric pulse broadening induced by third order dispersion, and ways to compensate for that [173]. The scheme has also been extended to study the waveform and transmition of phase modulated data [174]. Dual optical combs were also used in the characterization of RF signals, by ‘channelizing’ the RF spectrum, as demonstrated in [175].

The scheme proposed by Fontaine [127], which used a comb to detect coherent pieces of the optical spectrum and then splicing them together, has been used to characterize broadband optical communication signals, as discussed in Section 4.6. However, optical combs can also be used to generate and detect wavelength division multiplexed signals in optical communications, as reviewed in [176]. Mazur et al., demonstrated how a comb used to generate WDM channels in a transmitter could be coherently regenerated in the receiver, either by transmitting two pilot tones [177] or a single [178] pilot tone. By regenerating the comb from the co-transmitted pilot and using it as local oscillator, 50 wavelength channels could be coherently detected without requiring any digital phase-tracking algorithms. Modulation formats as complex 128-QAM were demonstrated with these schemes [178].

In addition, the fact that frequency combs have a well defined phase relation among the lines can be exploited in data transmission in various ways. When using two free-running combs as transmitter and local oscillator, the beating phase, which is dominated by the laser phase noise at the two lasers used to generate the combs, will be well correlated among the wavelength channels [179]. This can be used to reuse the extracted phase noise from one channel on several other channels, which will then have significantly less complex digital signal processing [180]. Alternatively, the retrieved phase can be jointly processed to increase the tolerance to nonlinear phase noise.

While the transmission schemes described above have been mainly demonstrated with electro-optic combs, microcombs have also been shown to work [181], provided the receiver comb is optically locked to two modes of the transmitter comb, as was carried out for electrooptic combs in [177].

Frequency combs have also supported the detection of optical orthogonal frequency domain modulation (OFDM), where a large number of microwave carriers are modulated onto an optical carrier. By using a local oscillator comb, the various carriers can be detected in parallel [182].

## 6. Summary and Outlook

Laser frequency combs provide a grid of optical frequencies whose location can be set with the accuracy and stability given by atomic frequency references. We have illustrated how these characteristics enable the calibration of continuously tunable lasers used in swept wavelength interferometry and enhanced signal to noise ratio in Fourier transform spectroscopy and linear spectral interferometry. Phase coherence can be maintained between combs, facilitating so called dual comb interferometry.

Chip scale frequency comb sources derived from mode locked lasers [183], electro-optic combs [184] or microcombs [185] all feature a line spacing much larger than what is possible with standard passively mode locked lasers. In the context of linear interferometry, this results in a potentially higher power per line (assuming equal average comb power), higher acquisition rates and ease in resolving comb lines. These are particularly interesting features in the context of dynamic spectroscopy [145] or when applying linear interferometric techniques in new applications, such as lidar [186,187,188] and telecommunications [127,180], where target objects or signals are rapidly varying in the sub-microsecond time scales. However, the spectral sampling resolution when using chip scale comb sources is naturally coarser, and care must be taken when using these comb sources in the context of, e.g., static gas spectroscopy. Tuning the comb parameters, such as offset [125,126] or line spacing [119,120], offer exciting technological prospects to overcome these limits.

In terms of complex photonic integration, the possibilities enabled by rapid electro-optic [189] and piezoelectric control [190], the generation of low linewidth lasers in ultralow loss planar technologies [190,191] and broadband nonlinear optics [189,192,193] could offer new prospects in reaching the mid-infrared [194], the realization of ultrafast tunable lasers on chips [195] and novel spectroscopy modalities enabled by massive space parallelization [196]. The field is broad and rapidly evolving, and new applications will be unleashed as heterogeneous integration technologies mature [197,198] and we gain understanding of the fundamental limits in phase coherence in chip scale comb sources [199,200,201,202]. 

## Abbreviations

The following abbreviations are used in this manuscript:

FTS	Fourier transform spectroscopy
FSR	Free spectral range
TD-SI	Time domain-spectral interferometry
SDI	Spectral domain interferometry
SWI	Swept-wavelength interferometry
OFDR	Optical frequency domain reflectometry
CEO	Carrier envelope offset
MZI	Mach Zehnder interferometry
AWG	Arrayed waveguide grating
DSP	Digital signal processing
ADC	Analog to digital converter
DRS	Double resonance spectroscopy
ISRS	Impulsive stimulated Raman scattering
OTDR	Optical time domain reflectometry
SS	Swept source
FD	Fourier domain
WDM	Wavelength division multiplexing
OFDM	Orthogonal frequency domain modulation
RF	Radio frequency
CW	Carrier wave
DUT	Device under test
SNR	Signal to noise ratio
OSNR	Optical signal to noise ratio
MLL	Mode-locked laser
FDML	Fourier domain mode locking
DCI	Dual-comb interferometry
SWI	Swept-wavelength interferometry
DC-SWI	Dual comb swept wavelength interferometry
EO	Electrooptic
EOM	Electrooptic modulator
PM	Phase modulator
IM	Intensity modulator
MZM	Mach–Zehnder modulator
OCT	Optical coherence tomography
ECL	External cavity laser
FFT	Fast Fourier transform
FFP-TF	Fiber Fabry–Perot filter
FFP-FC	Frequency comb fiber Fabry–Perot filter
FSR	Free spectral range
DAQ	Data acquisition
